# Ring Chromosome 20 Syndrome: Genetics, Clinical Characteristics, and Overlapping Phenotypes

**DOI:** 10.3389/fneur.2020.613035

**Published:** 2020-12-08

**Authors:** Angela Peron, Ilaria Catusi, Maria Paola Recalcati, Luciano Calzari, Lidia Larizza, Aglaia Vignoli, Maria Paola Canevini

**Affiliations:** ^1^Human Pathology and Medical Genetics, ASST Santi Paolo e Carlo, San Paolo Hospital, Milan, Italy; ^2^Child Neuropsychiatry Unit - Epilepsy Center, Department of Health Sciences, ASST Santi Paolo e Carlo, San Paolo Hospital, Università Degli Studi di Milano, Milan, Italy; ^3^Division of Medical Genetics, Department of Pediatrics, University of Utah School of Medicine, Salt Lake City, UT, United States; ^4^Laboratory of Cytogenetics and Molecular Genetics, Istituto Auxologico Italiano IRCCS-Istituto di Ricovero e Cura a Carattere Scientifico, Cusano Milanino, Milan, Italy; ^5^Bioinformatics and Statistical Genomics Unit, Istituto Auxologico Italiano IRCCS-Istituto di Ricovero e Cura a Carattere Scientifico, Cusano Milanino, Milan, Italy

**Keywords:** ring chromosome 20 syndrome r(20), ring chromosomes, mosaicism, cytogenetics, karyotype, seizures, rare disease, epilepsy

## Abstract

Ring chromosome 20 [r(20)] syndrome is a rare condition characterized by a non-supernumerary ring chromosome 20 replacing a normal chromosome 20. It is commonly seen in a mosaic state and is diagnosed by means of karyotyping. r(20) syndrome is characterized by a recognizable epileptic phenotype with typical EEG pattern, intellectual disability manifesting after seizure onset in otherwise normally developing children, and behavioral changes. Despite the distinctive phenotype, many patients still lack a diagnosis—especially in the genomic era—and the pathomechanisms of ring formation are poorly understood. In this review we address the genetic and clinical aspects of r(20) syndrome, and discuss differential diagnoses and overlapping phenotypes, providing the reader with useful tools for clinical and laboratory practice. We also discuss the current issues in understanding the mechanisms through which ring 20 chromosome causes the typical manifestations, and present unpublished data about methylation studies. Ultimately, we explore future perspectives of r(20) research. Our intended audience is clinical and laboratory geneticists, child and adult neurologists, and genetic counselors.

## Introduction

Ring chromosomes (RCs) are rare genetic events that result from an intra-chromosomal fusion (1). Constitutional rings have been detected in all human chromosomes, and their prevalence is estimated to be between 1 in 30,000 and 1 in 60,000 live births (1, 2). They can be associated with a clinical phenotype—called ring (chr) syndrome—or have little to no clinical consequences depending on the chromosome involved.

Two major types of RCs have been described: (1). 46,(r), where a full-length or an unbalanced ring replaces one of the normal linear homologs; (2). 47,+(r), where a small supernumerary chromosome containing pericentromeric chromatin is present in addition to the normal chromosomal set (3). In both cases the cell line carrying the RC may coexist with the normal cell line in a mosaic condition.

Among RCs, ring chromosome 20 [r(20)] is one of the most intriguing and less understood. r(20) was first described in 1972 in two children with seizures and behavioral problems with or without intellectual disability (ID) (4, 5), and a ring chromosome 20 syndrome was proposed by Herva et al. 5 years later (6). To date, about 200 pediatric and adult individuals with r(20) syndrome have been reported in the literature (Supplementary Table 1).

Although r(20) syndrome has a distinctive and recognizable epileptic phenotype, we acknowledge that it is not well-known among clinical geneticists and neurologists and may therefore be underdiagnosed, especially in the genomic era. In this review we will address the genetic and clinical aspects of r(20) syndrome and discuss differential diagnoses and overlapping phenotypes, providing the reader with useful tools for clinical and laboratory practice. We will present some unpublished data and the results of a comprehensive literature review. Our intended audience is clinical and laboratory geneticists, child and adult neurologists, and genetic counselors.

## Genetics of r(20) Syndrome

### Ring Chromosomes

Supernumerary RCs are often small and include the pericentromeric sequences (7), whereas non-supernumerary RCs (on focus herein) tend to be less unbalanced or even complete. Losses and/or gains of genetic material can be present depending on the RC formation mechanism. At least three mechanisms generating RCs have been proposed (8): (1) Double-strand breaks; (2) Telomere junction; (3) Inv dup del rearrangements (Table 1). RCs involving chromosome 20 resulting from any of the three proposed mechanisms have been described.

**Table 1:** Proposed RCs formation mechanisms.

**Mechanism**	**Break and fusion**	**Telomere/subtelomere junction**	**inv dup del rearrangement**
Predisposing event	Double-strand breaks (after exposure to ultraviolet radiation)	Critical shortening of telomere repeats [Surace et al. (9)]	U-type recombination. During meiosis I parental chromosomes may recombinate at microhomology regions. The result is a dicentric chromosome that undergoes asymmetric breakage with consequent formation of a monocentric linear rearranged chromosome with a terminal deletion and an inverted duplication
Description	An inefficient DSBs repair with fusion of two unstable chromosome ends or fusion of an unstable chromosome end with the opposite telomeric end	Junction of telomeric or subtelomeric sequences of the p and q arms of the same chromosome	Fusion of a broken rearranged chromosome end (originated as the consequence of an intra-chromosomal U-type recombination) and the opposite arm of the same chromosome
Genetic imbalances on the resulting RC	Loss of genetic material on the p and/or q arm whose extent depends on the distance between the break and the telomere	No loss of genetic material is present, with the exception of the common telomeric sequences that may be missing in some cases	Variable combination of losses and gains within the arm involved in the U-type recombination
Schematic representation	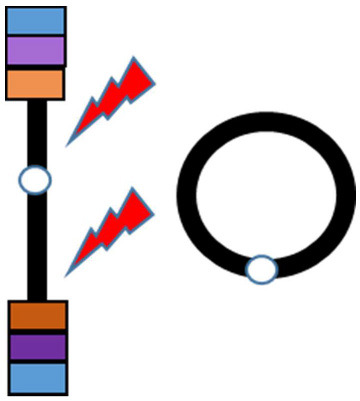	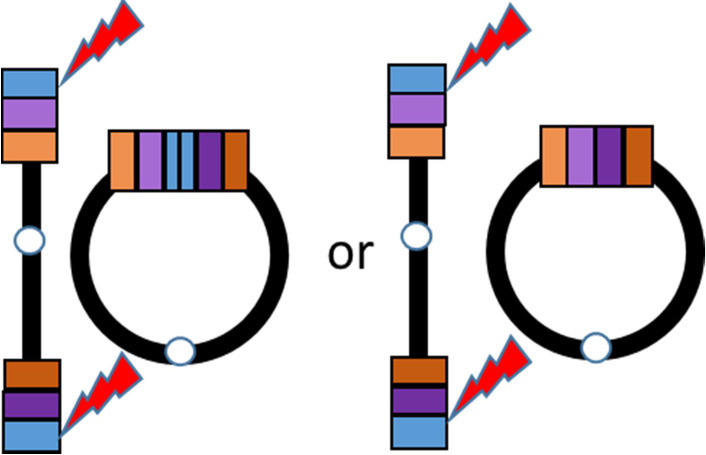	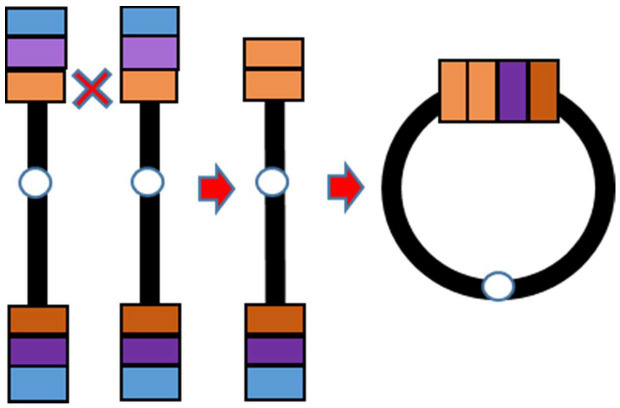
Examples in literature	r(20): Conlin et al. (10) (pts 22, 24, 26, and 28) r(3), r(10), r(13), r(15), r(18), r(22): Guilherme et al. (11) (pts 1–5, 8–11, 13,14)	r(20): Giardino et al. (12) r(20): Conlin et al. (10) (pts 1–21) r(14) and r(22): Guilherme et al. (11) (pts 7, 12) r(17): Surace et al. (9)	r(20): Conlin et al. (10) (pts 26 and 27) r(13): Guilherme et al. (11) (pt 6) r(7) and r(13): Rossi et al. (13)

*For each mechanism a schematic description is provided for the normal (left) linear chromosome and the derived RC (right). Red flash: break event; red cross: U-type exchange event; light blue boxes: common telomeric repeats; violet boxes: specific p/q arm subtelomeric sequences; brown boxes: inner arm specific sequences*.

Because of their circular shape, RCs are unstable in dividing cells and tend to be lost, duplicated, or rearranged during mitosis. The observation of dicentric, duplicated, re-opened, and broken RCs by Barbara McClintock in maize cells dates back to 1938 (14) and anticipates the observation of the behavior of RCs in mammalian and human cells during mitosis. A RC is considered unstable if it rearranges in more than 5% of cells (15). Ring instability varies in each single case, as described by Guilherme et al. (11) who studied 14 RCs carriers and observed that instability ranged from 4 to 16.3% on 300 analyzed cells.

Some recurrent clinical features (i.e., pre- and post-natal growth delay and mild to moderate ID) were initially observed in RCs carriers independently of the chromosomal origin, and a “ring syndrome” was proposed in the past (16). The phenotype was thought to be caused by the lower growth and higher level of cell death within RCs due to their intrinsic instability. However, subsequent studies showed that growth delay is not a recurrent feature in several ring-associated syndromes and a “ring syndrome” is unlikely to exist (1, 17). When supernumerary RCs are present, the phenotype is generally attributable to the increased dosage of the genes that are located on the RC and are present in three or more copies, while the phenotype of non-supernumerary RC carriers is strongly influenced by the haploinsufficiency of deleted genes. Uniparental disomy (UPD) can be an additional cause of the phenotype, should imprinted genes be located on the rearranged chromosome, and mutant alleles responsible for autosomal recessive diseases be unmasked.

### Ring Chromosome 20 Syndrome

r(20) syndrome is a rare genetic disorder characterized by a ring chromosome 20 replacing a normal chromosome 20. It is diagnosed by means of conventional cytogenetics (karyotyping). The ring chromosomes have been reported in different tissues and have been seen prenatally in both the amniotic fluid and chorionic villi samples, as well as in postnatal peripheral blood, bone marrow, and fibroblasts (Supplementary Table 1). It seems to be more frequent in females (60%) than in males (40%) (*p* < *0.001*).

Patients with a supernumerary r(20) have also been described in about 10 studies. They will not be discussed in this review since supernumerary r(20) is considered a different syndrome with a distinct phenotype (18). Updated information about supernumerary r(20) are available at http://cs-tl.de/DB/CA/sSMC/0-Start.html.

In 2011 cytogenetics and molecular genetics analyses performed on 28 patients with r(20) syndrome highlighted the presence of two distinct groups carrying a non-supernumerary r(20) chromosome (10):

**(A) Non-mosaic r(20)**: The first group included patients with r(20) in all cells (100%) (Supplementary Table 1). In these patients the r(20) did not show telomeric repeats at the ring junction and was characterized by microdeletions of at least one variably-sized subtelomeric sequence with no recurrent breakpoints (10). These findings were consistent with the r(20) originating during meiosis through a break-and-fusion mechanism (Table 1). To date a total of 26 non-mosaic r(20) patients have been reported in the literature. Information about sex is available for 18 individuals: 11 are males, and 7 are females. Although a male prevalence is suggested, additional data are needed to statistically confirm the sex-specific prevalence.

**Figure 1:**
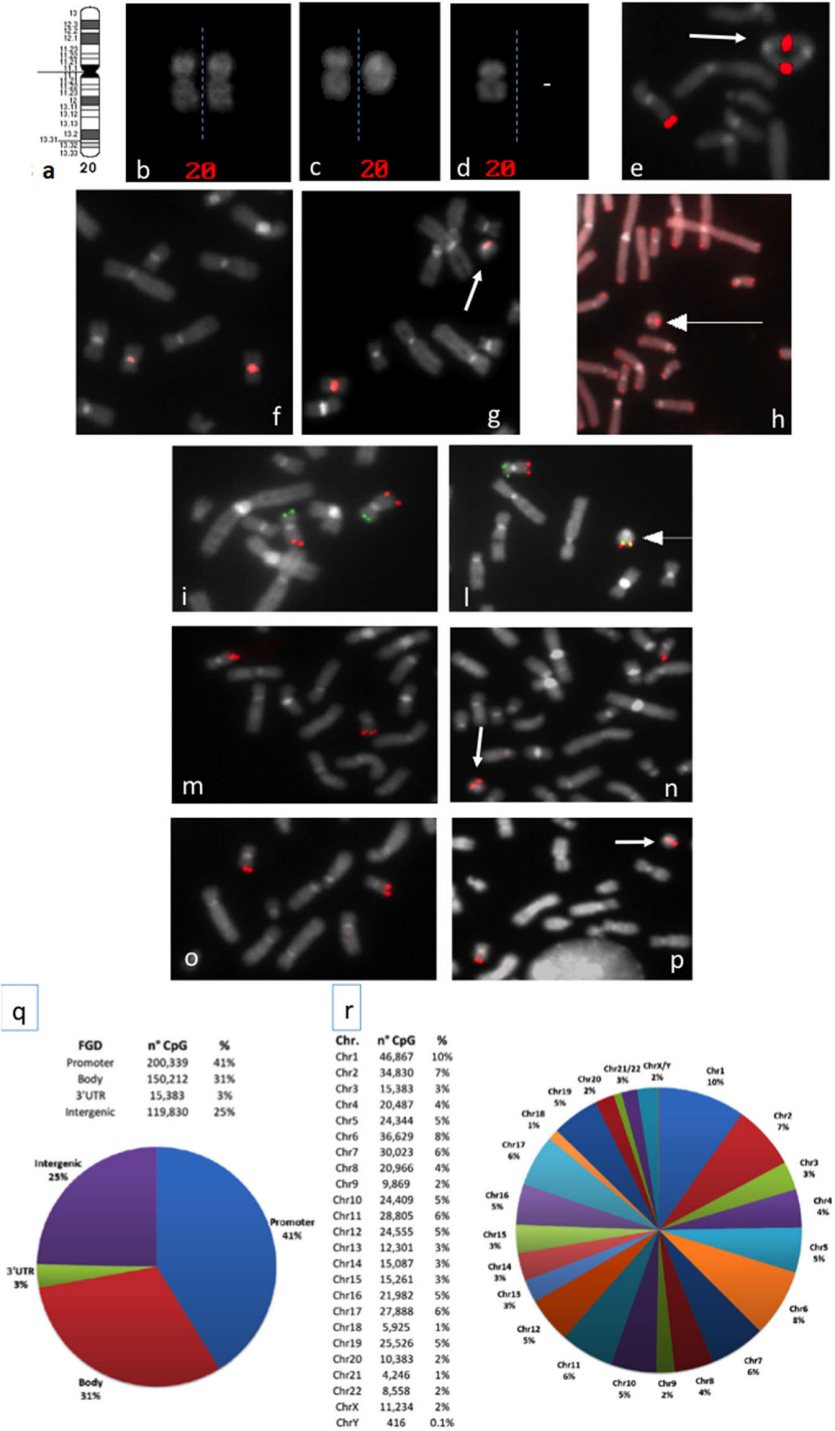
**(a)** Chromosome 20 ideogram; **(b–d)** QFQ-banded chromosomes 20: **(b)** normal chromosomes 20 homologs; **(c)** normal chromosome 20, left, ring chromosome 20, right; **(d)** chromosome 20 monosomy; **(e)** duplicated ring upon FISH experiments with the BAC probe RP11-939M14 mapping on 20q13.33 (red signals); **(f,g)**: FISH with ALF 20 FISH probe specific for chromosome 20 shows centromere heteromorphism (the intensity of the centromeric signal is different in the two homologs): **(f)** normal chromosomes 20, **(g)** ring 20 chromosome (arrowed) with the low intensity centromeric signal shown by one chromosome 20 of the normal cell line; **(h)** FISH with pantelomeric probe (red signals) shows common telomeric sequences on ring 20 chromosome (arrowed); **(i–l)** Subtelomeric arm-specific FISH probes (green: p arm, red: q arm) show the respective signals on **(i)** normal chromosomes 20 and **(l)** ring 20 chromosome (arrowed); **(m,n)** FISH with BAC probe RP11-939M14 (red) on **(m)** normal chromosomes 20 and **(n)** ring 20 chromosome (arrowed) demonstrates the absence of *CHRNA4* deletion; **(o,p)** FISH with BAC probe RP11-358D14 (red) on **(o)** normal chromosomes 20 and **(p)** ring 20 chromosome (arrowed) demonstrates the absence of *KCNQ2* deletion; **(q,r)** Summary of the 450K DNA methylation array: **(q)** classification of CpG sites according to the functional genomic position: promoter, body, 3′UTR, and intergenic (FGD, Functional Genomic Distribution); **(r)** distribution of CpG sites among chromosomes.

**(B) Mosaic r(20)**: The second group included mosaic patients with a normal cell line and a cell line containing the r(20), ranging from <1 to 99% (Supplementary Table 1 and Figures 1a–d).

In 2010 Giardino et al. cytogenetically re-analyzed five previously reported r(20) patients (19, 20) and observed that the r(20) percentage maintained a fairly stable trend across time (Supplementary Table 1). Clinicians and laboratory providers should therefore keep in mind that a high number of metaphases should be counted when r(20) syndrome is suspected. In mosaic patients the r(20) maintained intact subtelomeric and telomeric sequences, and no genomic imbalances of the chromosome were detected (Figures 1h,i,l). Based on this evidence, post-zygotic telomere fusion is thought to be the most probable mechanism for ring formation (Table 1).

**Figure 2:**
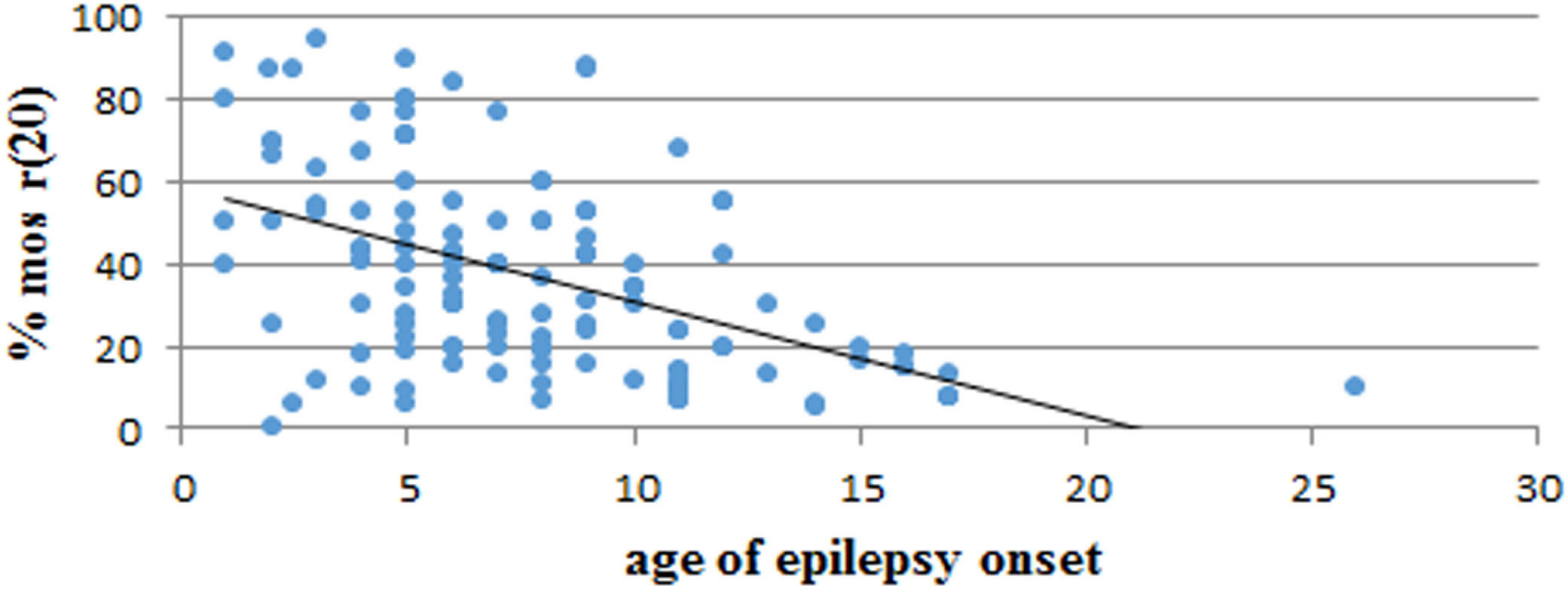
Graph showing the correlation between the percentage of r(20) mosaicism and the age of epilepsy onset in reported patients with mosaic r(20). The linear interpolation is indicated.

**Figure 3:**
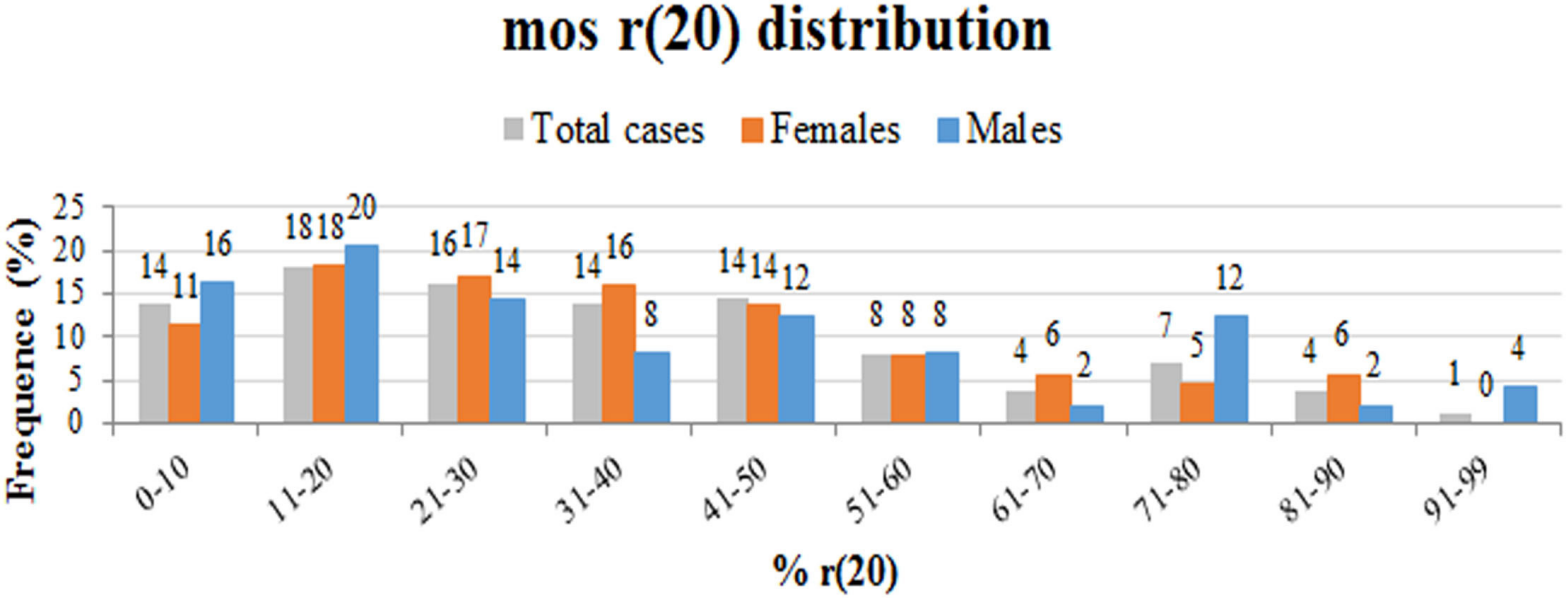
Graph showing the frequency of different categories of r(20) mosaicism (from lowest to highest) in all described r(20) patients (gray), in r(20) females (red), and in r(20) males (blue). The percentage of r(20) in each category is indicated above each column.

We performed a retrospective analysis of all patients from the literature and found more than 150 patients in the mosaic r(20) group. Females (64%) seem to be more frequently affected than males (36%) (*p* < *0.0001*) (Supplementary Table 1). Our analysis also confirms that the percentage of cells containing the ring chromosome inversely correlates with the age of seizure onset (Figure 2), in line with the literature (1, 10, 21, 22). The distribution of patients in the mosaic group with a different percentage of r(20) cell population is similar between females and males (Figure 3). The results of the analysis regarding the clinical manifestations are reported in the “clinical characteristics” section.

Despite the classification of r(20) patients in two groups (10), three individuals have been reported to carry a r(20) with characteristics that overlap between the two groups, as their r(20) was in a mosaic state but showed terminal imbalances on molecular analyses (22–24). In line with this observation, Colin et al. reported on two patients with additional genomic alterations of the r(20) chromosome. In particular, the inverted orientation of the detected duplication in one patient is in accordance with an origin mediated by the inv dup del rearrangement mechanism (Table 1) (10). Therefore, subgrouping of r(20) patients may be less strict than previously proposed (10), suggesting that the r(20) structure is more complex than so far envisaged. As a matter of fact, conventional cytogenetic analysis—which undoubtedly represents the gold standard for diagnosis—has been integrated with the characterization of r(20) by molecular analyses only in a few studies thus far (summarized in Table 2 and in Supplementary Table 1), and should be implemented to elucidate this matter.

**Table 2:** Cytogenetics, cytogenomics, and molecular techniques used to study ring chromosome 20 [r(20)].

**Method**	**Advantages/aim**	**Limitations**	**References**
Karyotype on peripheral blood (Figures 1b–d)	r(20) identification Analysis extended up to 200 metaphases to detect low-level mosaicism	Unrecorded: - chromosomal aberration <10 Mb - low mosaicism level - tissue-specific mosaicism	Atkins et al. (4) (first description)
Karyotype on skin fibroblasts or other tissues	r(20) identification and/or confirmation of ring 20 syndrome in case of undetected ring 20 on peripheral blood Multi-tissue estimation of mosaicism. Analysis extended up to 200 metaphases to detect low-level mosaicism		Faed et al. (5) (first report); Back et al. (25), Zou et al. (26), Giardino et al. (12), Cappanera et al. (27), Elens et al. (22)
Prenatal karyotype analysis (chorionic villi, amniotic fluid)	Precocious diagnosis of ring chromosome 20 syndrome, with consequent genetic counseling and follow up		Giardino et al. (12), Cignini et al. (28)
FISH with pantelomeric probe (Figure 1h)	Assess if common telomeric sequences are present/absent.	Lack of signal on the ring does not determine deletion extent.	Zou et al. (26), Elghezal et al. (29), Giardino et al. (12), Unterberger et al. (30), Tayama et al. (31)
FISH with 20p-20q subtelomeric probes (Figures 1i,l)	Assess if a subtelomeric deletion is present/absent		*Idem*; de Falco et al. (23), Herrgård et al. (32), Cappanera et al. (27), Gahr et al. (33), Inal et al. (34)
FISH with probe specific for chromosome 20 centromeric sequences (Figures 1f,g)	Identification of chromosome origin of the RC Evaluation of alphoid-specific heteromorphism of r(20) and its linear homolog Detection of low chromosome 20 mosaicism for a monosomic cell line	Deletion/duplication cannot be detected	Giardino et al. (12), Kamoun et al. (35)
FISH with whole chromosome 20 painting probe	Detection of other chromosome regions on r(20) (low resolution)	Deletion/duplication cannot be detected	Elghezal et al. (29), Zou et al. (26), Cabras et al. (24), Tezer et al. (36)
BAC FISH on *CHRNA4* and *KCNQ2* candidate genes (Figures 1m–p)	Detection of deletions of candidate genes. (resolution higher than karyotype)	Limited to the targeted sequence(s). Resolution lower than CMA microarray	Zou et al. (26), Elghezal et al. (29), Giardino et al. (12); Cappanera et al. (27), Kamoun et al. (35)
Segregation analysis of polymorphic loci	Exclusion of whole or segmental UPD20	Tissue specific UPD and low-level mosaicism cannot be detected	Giardino et al. (12)
**Chromosomal microarray**			
Array-CGH (Resolution from 30 to 0.6 Mb)	Identification of CNVs on chromosome 20 and in the whole genome	Tissue specific and low-level mosaicism cannot be detected	Giardino et al. (12); Cabras et al. (24), Rodan al. (37), Corrêa et al. (38)
SNP-array (Resolution from 4.2 to 8.2 kb)	Identification of CNVs, UPD, and homozygosity regions on chromosome 20 and in the whole genome		Conlin et al. (10), Unterberger et al. (30)
Array-based genome-wide methylation analysis array (Human Methylation450 BeadChip kit, Illumina)	Evaluation of the methylation level of CpGs in the whole genome in r(20) patients compared to normal controls	Tissue-specific and low-level epimutation mosaicism cannot be detected	Calzari L. [patients from Giardino et al. (12)]; present data (Figures 1q,r)

### r(20) Inheritance

**Figure 4:**
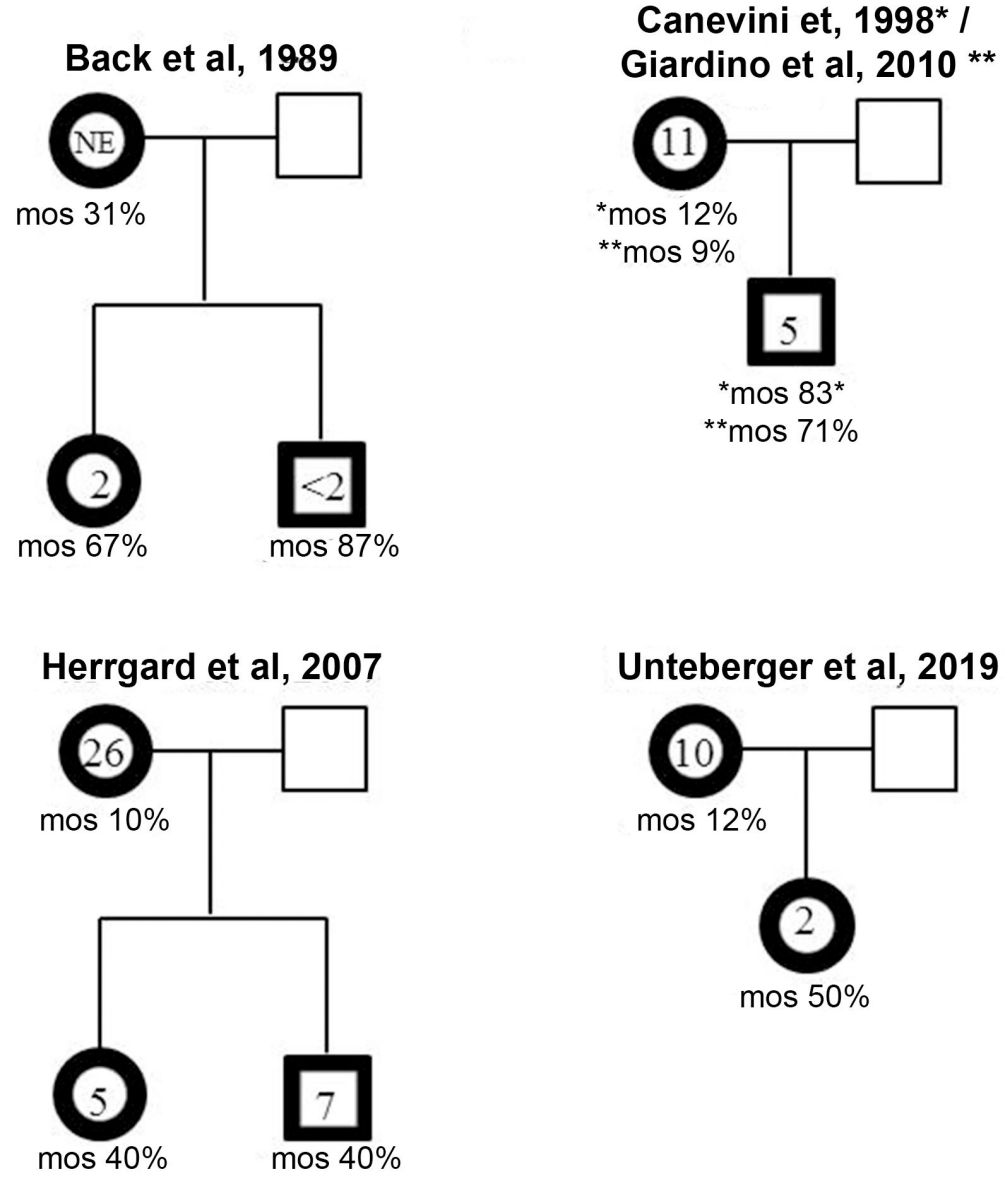
Mosaic-to mosaic maternal transmission of r(20) in four reported family trees. The age of epilepsy onset and the percentage of r(20) mosaicism are indicated within and below the affected individuals (bold framed symbols).

Like other ring chromosomes, r(20) is generally unstable and occurs sporadically in most patients. However, four familial cases have been reported thus far. In all the families a mosaic mother transmitted r(20) to the offspring in a mosaic state (Figure 4) (12, 19, 25, 30, 32) (Supplementary Table 1). A familial mosaic-to-mosaic transmission has been reported for supernumerary r(19), r(20), and r(17) as well (9, 39, 40). In all the familial cases, the percentage of cells containing the ring was higher in the offspring compared to the mothers and correlated with an earlier epilepsy onset (9, 12, 19, 30, 32, 39, 40).

The predominance of maternal transmission has been noted since 1991 by Kosztolanyi, who found that the ring chromosome had a paternal transmission in only two out of 23 families with inherited autosomal ring chromosomes (41). A possible explanation is represented by the fact that female fertility seems to be less compromised by ring chromosomes than male fertility. This is because the meiotic cell cycle checkpoints are less stringent in females than in males (42). Further evidence is provided by the maternal segregation of the ring chromosome in more than one child from both families reviewed by Kosztolanyi, and in two of the four above mentioned families with r(20). Male ring carriers often experience reduced fertility as indicated by a low sperm count (43–46).

Moreover, the recognized familial ring transmission is likely underestimated, as low-level mosaicism for a ring chromosome—especially a small one—may be easily overlooked, as attested by the report of a healthy father of a patient with r(14), who exhibited the ring chromosome in three out of 288 cells analyzed (47).

### r(20) Mosaic Origin

Four hypotheses have been proposed to explain both the mosaicism in r(20) patients and the mosaic-to-mosaic transmission in familial cases:

1. Inheritance of the r(20) by the zygote and early loss of the ring in a subset of cells with consequent monosomy rescue by re-duplication of the normal chromosome 20. The cell population without the ring chromosome exhibits UPD;
2. Inheritance of a supernumerary r(20) by the zygote and early random trisomy rescue by UPD with mosaic state;
3. Inheritance of the r(20) by the zygote with subsequent opening of the unstable ring chromosome in one of the post-zygotic mitotic divisions;
4. Inheritance of the normal chromosome 20 by the zygote with subsequent chromosome 20 closing during post-zygotic mitotic divisions driven by instability factors, such as telomere shortening (1, 10, 12, 30).

Unlike hypotheses 1 and 2, the offspring predicted by hypotheses 3 and 4 implicates biparental inheritance.

UPD has been demonstrated in patients who are mosaic for a normal cell line and a cell line carrying r(21) (48, 49). This finding was interpreted as a mechanism compensating the loss of the abnormal chromosome by duplication of its normal homolog. The compensatory UPD mechanism has been found effective in induced pluripotent stem cells (iPSCs) reprogrammed from fibroblasts of ring chromosome patients, as the cell-autonomous correction by loss of the unstable ring chromosome and duplication of the normal homolog was observed in the *in vitro* model (50). However, at least two studies argue against hypotheses 1 and 2, ruling out UPD by microsatellites and SNP-array analyses in blood lymphocytes of individuals with r(20) (12, 30), as observed in r(14) as well (51).

In addition to the haplotype analysis, Giardino et al. also exploited FISH analysis using a 20-specific alphoid probe able to visualize signal intensities that are dependent on the different number of alphoid repeats. The application of this tool allowed to differentiate the linear and the r(20) chromosomes in some patients carrying this centromeric polymorphism (12) (Figures 1f,g). Interestingly, the results suggested that the circularized chromosome 20 is the same that is not circularized in the normal cell line (12). This indicates that r(20) patients have two cell lines sharing two chromosomes 20 that are genetically different only in a morphologically detectable alphoid polymorphism. A different epigenetic conformation of the ring chromosome responsible for a differential gene expression compared to that of the linear chromosome is a challenging possibility that remains to be definitely proved or excluded.

Summing up, the evidence so far available argues against hypothesis 2, whereas it does not conflict with hypotheses 3 and 4. However, different studies proposed conflicting interpretations even for the latter pathomechanisms (30). For instance, Surace et al. (9) reported on a familial case of r(17) transmitted from mother to daughter, who were both mosaic for a prevalent normal cell line and a minor cell line with r(17) shown by FISH analysis to be complete. By quantitative FISH analysis on either normal or ring metaphases of the proband, chromosome-specific telomere lengths resulted significantly shorter than in controls, while the telomere length of both the normal chromosome 17 and the r(17) in the proband's mother was comparable to that of age-matched controls. Segregation from parents to proband of informative chromosome 17 STRs was consistent with biparental inheritance. Based on these results, the authors hypothesized that the r(17) in the abnormal cell line had been inherited as a normal chromosome 17 that, having critically short telomeres, was predisposed to close giving rise to the ring. On the contrary, Speevak et al. (39) suggested postzygotic ring opening as the most probable explanation for mosaic r(19). The Authors performed dilution cloning in cell cultures, with subsequent analysis of microsatellite markers to study a prenatally inherited ring 19 chromosome, with preserved telomere repetitive sequences. The Authors found normal biparental inheritance in a subclone with normal karyotype and an increasing percentage of cells with normal karyotype in later steps, which was consequent to the loss of r(19).

Further indirect evidence in favor of the ring-opening hypothesis has been recently provided (30). The Authors hypothesize that during meiotic cell division, transmission of a small ring chromosome can pass through safely only if the transmitted chromatid is not involved in sister chromatid exchange at the meiosis prophase. Even a single crossover can lead to a double-sized dicentric chromatid, which—being unstable—will likely be disrupted during subsequent cell divisions. Conversely, transmission of a linear chromosome would allow correct recombination between non-sister chromatids. The authors applied SNP-array genotype analysis and observed that the r(20) was transmitted without recombination in the reported family. Similarly, Giardino et al. (12) demonstrated mother-to-child transmission of the r(20) mosaicism without recombination by means of microsatellite analysis. These pieces of evidence together argue in favor of familial ring transmission and subsequent opening to create the normal cell line.

Whether mosaicism is due to a postzygotic linear chromosome closing or to a ring chromosome opening is still an outstanding question.

### r(20) Pathophysiology

Several hypotheses have been raised to explain the clinical phenotype associated with r(20) syndrome: (1) deletion of candidate genes close to 20p and 20q telomeres; (2) epigenetic silencing of candidate genes near the telomeres; (3) deleterious effect of ring instability on cellular proliferation and function; and (4) compensatory UPD. As mentioned above, the last mechanism has been excluded by molecular genetic analyses on r(20) patients (12, 30).

The main candidate genes for the r(20) syndrome phenotype are *CHRNA4* (acetylcholine receptor, nicotinic, alpha 4—OMIM #118504) and *KCNQ2* (potassium voltage-gated channel subfamily Q member 2 – OMIM #602235) on 20q13.3, located 1 Mb from the telomere. Pathogenic variants or deletions of *CHRNA4* are associated with autosomal dominant frontal lobe epilepsy (ANDFLE; OMIM #600513), whereas pathogenic variants of *KCNQ2* cause benign familial neonatal seizures (BNFS, OMIM 602235). Another gene on 20q13.3, located 450 kb from the telomere and associated with epilepsy, is *DNAJC5* (DnaJ Heat Shock Protein Family (Hsp40) Member C5—OMIM # 611203). Pathogenic variants of *DNAJC5* cause an autosomal dominant form of adult-onset ceroid lipofuscinosis, a rare hereditary neuropsychiatric disorder characterized by neuronal loss and seizures. Since the type of epilepsy associated with these three genes is different from that observed in r(20) patients, it has been hypothesized that the concomitant deletion of these or other candidate genes near the telomere during r(20) formation might be responsible for the clinical phenotype of the r(20) syndrome.

However, deletions in r(20) have been detected only in few affected individuals with different breakpoints, not always including *CHRNA4, KCNQ2, DNAJC5*, or other genes on 20q13.3 (Figures 1m–p). In addition, patients carrying terminal deletions of the short or long arm of a linear chromosome 20 do not show the same epilepsy of r(20) syndrome patients (52–54).

Therefore, the molecular etiology of the r(20) syndrome phenotype could be due to a different causative mechanism rather than deletions, possibly linked to the structure of the ring chromosome itself. However, even if patients with different ring chromosomes such as r(14) or r(17) manifest epilepsy, their seizures differ from those of r(20) patients (9, 55). Moreover, non-supernumerary r(2) and r(4) patients do not exhibit epilepsy (56, 57), suggesting that the phenotype is likely dependent on the specific chromosome involved.

Another hypothesis implies that the phenotype may be due to gene silencing effect of telomeric sequences in the ring chromosome. Telomere length varies from one individual to another mainly because of genetic factors inherited from the parents, paternal age at conception, and environmental factors (58–60). This data confirm the concept of a partially genetically inherited telomere length. Telomere length may influence the expression of genes that are close to the subtelomeric regions through a telomere positioning effect that depends on both the telomere length and the spreading of telomeric heterochromatin to nearby genes. Telomeric chromatin marks can spread and repress gene expression up to 100 kb from the telomere itself with a more pronounced effect when telomeres are long (60–62). In ring chromosomes—hence in r(20) syndrome—telomere-to-telomere fusion may cause an increasing silencing effect on genes close to the telomeres, which would be manifested by their down-regulation. Consistent with this hypothesis, the downregulation of genes located in the subtelomeric regions of r(14) and r(17) chromosome patients has been demonstrated by Real-Time Q-PCR integrated with other methods (9, 11, 63).

To explore this hypothesis, we have performed a preliminary genome-wide DNA methylation analysis through the Infinium HumanMethylation450 BeadChip array in previously investigated and two new r(20) patients (24, and unpublished patients). The 450K array-based platform is designed to test over 450,000 CpG sites distributed along all chromosomes and mainly covering the regulatory regions (promoters) of more than 20,000 coding genes (Figures 1q,r) (64). We set up a customized analysis pipeline to search for extreme aberrant methylation values (Stochastic Epigenetic Mutations—SEM) at a single case level (65), by comparing each r(20) methylation profile to that obtained from a control group of 112 healthy individuals. Enrichment analysis of SEM did not show any suggestive or highly shared epigenetic signature on either chromosome 20 or other chromosomes. No differences in methylation levels were found in the two main candidate genes *CHRNA4* and *KCNQ2*. However, given the relatively low coverage of subtelomeric regions on the 450K array, a refinement of the analysis by a custom-targeted methylation assay may be necessary to exclude epigenetic silencing of the genes located in the p and q subtelomeric regions in r(20) patients.

A third hypothesis about r(20) pathophysiology regards the intrinsic instability of ring chromosomes. A ring chromosome is unstable during cell division, due to its circular nature. If one or more crossovers occur between the ring chromosome and its normal homolog, additional abnormalities are generated, including duplicated rings or double rings (Figure 1e). Furthermore, ring loss occurs during cell division, resulting in chromosome monosomy (Figure 1d). The final result of chromosome instability is cellular apoptosis and growth delay, with several consequences on normal development. To date r(20) stability is controversial. Our retrospective literature analysis shows that only 12 out of 35 patients who were evaluated had secondary aberrations in more than 5% of the cells (Supplementary Table 1). This, supports the hypothesis that smaller ring chromosomes seem to be more stable than larger ones, since the lower the size of the chromosome the lower the probability of one or more crossover events to occur during meiosis. Nonetheless, the *in vitro* nature of these secondary changes should not be completely excluded (10).

## Clinical Characteristics of r(20) Syndrome

### Core Phenotype

Ring chromosome 20 syndrome in mosaic patients is characterized by a distinctive and recognizable epileptic phenotype and frequent—but not universal—cognitive decline and behavioral problems following seizure onset. Children with r(20) generally show normal development until seizure onset. The strict temporal relationship between the stormy onset of epilepsy and the progressive cognitive decline is consistent with the development of an epileptic encephalopathy (EE) (20).

**Figure 5:**
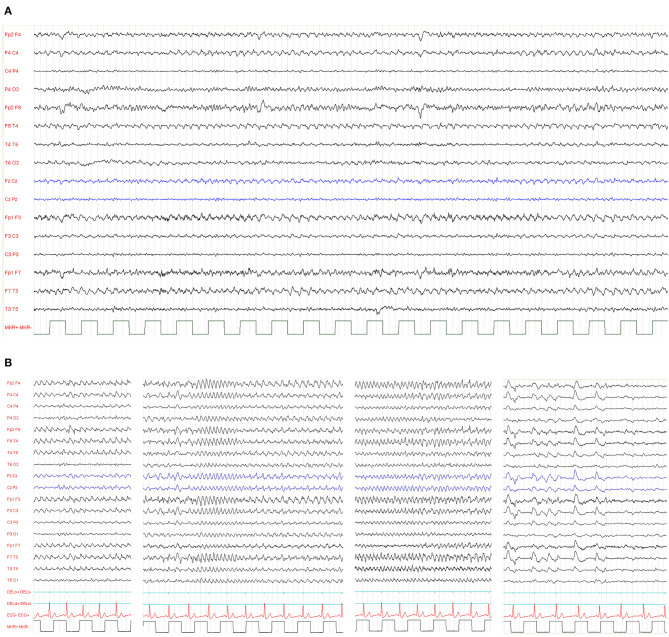
**(A)** Typical interictal electroencephalographic background activity in a 39-year-old patient exhibiting bursts of sharply contoured theta activity, with a peak frequency of 5 Hz, over the temporal regions. **(B)** Ictal EEG from a 26-year-old woman with ring (20) syndrome. Repetitive spikes occurred in both frontal regions, followed by 3–4-Hz slow waves and spike-and-wave complexes. Spike-and-wave complexes gradually lost the spike component with increasing frequency and became polymorphous. The NCSE episode lasted 40 min, and the breaks between these recordings are at seizure onset, after 10 min, 20 min, and at the end of the seizure, when she fell asleep. Her verbal response was impaired and slow. Complex mental action such as calculation was impossible.

The epileptic phenotype of r(20) syndrome is characterized by intractable focal seizures and non-convulsive status epilepticus (NCSE) (66, 67). The exhibits interictal electroencephalographic (EEG) background exhibits mild slowing or bursts of sharply contoured theta activity, with a peak frequency of 5 Hz, over the fronto-temporal regions (Figure 5) (19).

Based on clinical presentation and EEG characteristics, seizures in r(20) have been defined as refractory frontal lobe seizures, and three types of seizures have been documented (68):

1. **Nocturnal Seizures (Hyperkinetic or Hypermotor Seizures)** are characterized by waking up, staring, and mild tonic stiffening evolving into clonic movements of the face and of the extremities, followed by agitation and confusion (22, 69–71).
2. **Subtle Nocturnal Seizures** are expressed as minimal motor activity, such as subtle stretching, turning, or rubbing movements (69).
3. **Seizures With Impaired Awareness** are characterized by unresponsiveness, staring and confusion, with or without oral or motor automatisms, frightened expression, and focal motor symptoms including head turning (22, 67, 72).

Children with r(20) can experience terrific hallucinations even before the clear onset of their seizures (20, 70, 72). They have never been video-EEG recorded alone, but only in the initial phase of a focal motor seizure. However, we think that these events should be considered epileptic and diagnosed as ictal fear as a possible symptom of frontal lobe seizures that involve the limbic system.

**NCSE** is one of the key manifestations of r(20) syndrome. It consists of a prolonged confusional state of variable intensity and duration (66), associated with long-lasting slow waves with occasional spikes that are usually predominant over the frontal regions on the EEG (73). The particularity of r(20) is the recurrence of NCSE: patients with r(20) experience very frequent NCSE, which can present even daily. The clinical semiology during NCSE consists of altered state of vigilance, staring, loss of emotional facial expression, reduced spontaneous motor activity and speech production, with a slow response to questions. Associated motor symptoms, such as myoclonus, tonic posturing, oral automatisms, and frightened facial expression have been reported (66, 68). Frequent NCSE episodes in this syndrome might be related to the deregulation of the system(s) involved in seizure initiation and termination. Deficit of the striatal dopaminergic activity has been demonstrated using PET (74) and SPECT (75).

**Figure 6:**
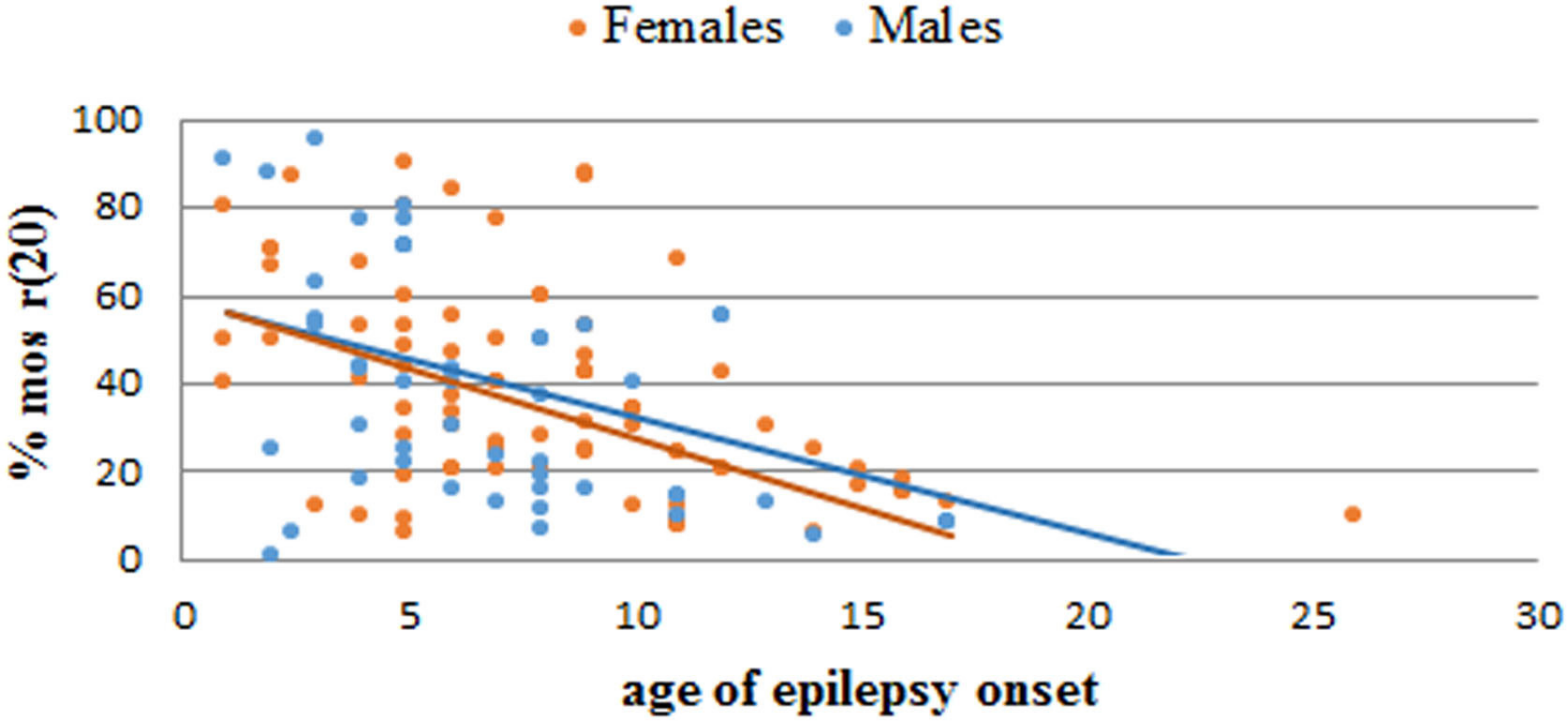
Graph showing the correlation between the percentage of r(20) mosaicism and the age of epilepsy onset in reported female (orange) and male (blue) patients with r(20). The linear interpolations are indicated.

Based on our literature review, the average age of epilepsy onset in r(20) syndrome is 7 years (8 yrs for females and 6 yrs for males). Most of the reported patients developed epilepsy at <10 years of age, with a trend toward a higher number of females in the range of 6–10 years and a higher number of males in the range of 0–5 years (Table 3). The age of epilepsy onset seems to similarly decrease with the increasing percentage of r(20) in males and females (Figure 6). Additional data need to be collected to statistically confirm this result. Also, non-mosaic patients present with seizures earlier in childhood compared to mosaic patients, and often show a more severe phenotype likely due to r(20) imbalances (10, 67, 72).

**Table 3:** Reported r(20) patients categorized by age at epilepsy onset (all patients and patients grouped according to sex).

**Age of epilepsy onset**	**r(20) patients**		
	**All patients (%)**	**Females (%)**	**Males (%)**
0–5	40.1	33.0	50.8
6–10	39.5	42.8	33.8
11–15	15.3	18.7	10.8
16–20	4.5	4.4	4.6
21–25	0	0	0
26–30	0.6	1.1	0

*Patients with age of epilepsy onset > 30 years are not reported*.

Recently, Vignoli et al. (67) demonstrated that epilepsy in ring (20) syndrome has an age dependent course. When epilepsy starts in childhood, very frequent nocturnal motor seizures or dyscognitive seizures associated with terrific hallucinations are the prominent manifestations, and often evolve into EE and NCSE. On the contrary, when epilepsy begins in adolescence the course is usually milder, with dyscognitive seizures and NCSE, but without cognitive decline.

With regard to behavioral problems and cognitive functioning, some individuals with r(20) may exhibit attention deficit, impulsivity and learning disabilities before seizure onset (22, 33, 72). In such individuals, reaching a chromosomal diagnosis can be even more difficult, also considering the fact that children with ID and behavioral problems are generally in charge of child psychiatrics rather than child neurologists. On the other hand, it has been clearly documented that in some children when epilepsy starts in childhood, the clinical presentation of r(20) should be interpreted as an epileptic encephalopathy (EE), because mental deterioration started and cognitive functioning partially regressed concomitantly with seizures and paroxysmal activity. Speech and executive abilities are frequently affected, resulting in apathy or hyperactivity, loss of social skills, obsessive behavior, psychosis, and autistic features (20).

Apart from epilepsy and cognitive decline, most patients with r(20) syndrome are otherwise healthy. Unlike other chromosomal abnormalities, r(20) individuals usually have normal pre- and post-natal growth parameters, and do not exhibit a distinctive facial appearance (1). However, non-specific minor facial anomalies have been occasionally described in some affected individuals (10). Although congenital malformations have been occasionally seen in r(20) patients, they are thought to be coincidental rather than part of the phenotype.

Little is known about the natural history of r(20). However, two subgroups of patients can be identified: a group with favorable outcome (no seizures, with or without medications), and a group with unfavorable course (refractory epilepsy with focal seizures and NCSE). The main determinant of the outcome is the age at seizure onset, having patients with later onset a better outcome (67).

### Diagnosis

The appearance of drug-resistant seizures in a normally developed child without dysmorphisms, birth defects, and structural anomalies on brain MRI does not usually suggest a chromosomal disorder. The challenge in diagnosing r(20) for clinicians is to recognize the typical electro-clinical characteristics and therefore request a karyotype instead of chromosomal microarray (CMA), which is frequently the first-tier test during genetic assessment. As stated above, the majority of patients present with mosaic r(20) on karyotype. For this reason, at least 100 metaphases should be analyzed in order not to miss the diagnosis (1).

Despite the rarity of these patients, the evaluation of r(20) individuals using prolonged video-EEG monitoring may allow shortening of the diagnostic odyssey in many, thanks to the recordings of the typical electro-clinical patterns (68).

Conventional brain MRI does not usually show structural anomalies in these patients. On the other hand, functional neuroimaging can help delineate the characteristics of brain involvement in r(20) syndrome. Indeed, PET, SPECT, and fMRI data are consistent with the notion that r(20) syndrome is associated with dysfunction of the frontal lobe network (76, 77) together with the basal ganglia (74, 75, 78).

### Management and Therapy

Individuals with r(20) often have drug-resistant seizures, and epilepsy represents the major burden for the patients and their families. In our experience, valproic acid and lamotrigine, often in combination, are generally the most effective antiepileptic drugs (AEDs) for treating seizures in r(20) (20). However, many patients continue to have drug-resistant epilepsy, and other AEDs have been reported to be effective, such as lacosamide (31, 79), ezogabine (80), and lithium (34). Some affected individuals have found beneficial effects from alternative treatments, such as ketogenic diet (81), while the efficacy of vagal nerve stimulation is controversial (26, 32, 67, 82). Finally, behavioral problems and anxiety can also be challenging to manage and may require specific treatments.

## Overlapping Phenotypes

Although r(20) syndrome can be considered an EE with a distinctive epileptic phenotype, some of the EEG characteristics and the semiology of seizures overlap with other conditions, often delaying the diagnosis. Also, given the absence of dysmorphic features, r(20) syndrome may not be suspected at the very beginning of seizures, and other epileptic syndromes may be erroneously diagnosed. Here we summarize the most common overlapping phenotypes of r(20) syndrome, and discuss similarities and differences.

### Epileptic Syndromes

Spike and waves in the EEG recordings, brief seizures characterized by staring and tonic clonic seizures that may be seen in adolescents and young adults with r(20) (19, 67) might resemble the onset of Generalized Genetic Epilepsies. Nevertheless, the other EEG features of r(20) mentioned above help in the diagnosis.

Gago-Veiga et al. (68) observed that all the patients with a confirmed cytogenetic diagnosis of r(20) syndrome had a triad of signs and symptoms (drug-resistant frontal lobe seizures, recurrent NCSE, and typical EEG), giving this electro-clinical triad a high sensitivity and negative predictive value (100%).

The Authors report that the differential diagnosis might be challenging especially with: (1) Frontal Lobe Seizures; (2) Rolandic Epilepsy treated with sodium channel blockers (NCSE during wakefulness); and (3) Lennox-Gastaut syndrome (LGS).

Frontal lobe seizures are an important seizure type in r(20). They are focal motor seizures, occur often during sleep, with sudden arousal, head-raising movements, frightened staring, and hyperkinetic movements such as bimanual automatisms or cycling (67). These features are often present in frontal lobe seizures of different etiologies (83), but the absence of NCSE and of the typical EEG features of r(20) syndrome are helpful in the diagnosis.

Moreover, Gago-Veiga et al. (68) reported a possible differential diagnosis in children with continuous spike and wave during slow sleep (CSWS) and recurrent NCSE during wakefulness, a clinical feature observed when incorrect treatment with sodium channel blockers is prescribed. In these cases, long runs of theta waves during sleep are generally observed.

The EEG pattern of subtle nocturnal frontal seizures is the same as that of nocturnal tonic seizures of LGS. However, the clinical features of the seizures are different, and predominantly characterized by subtle stretching, turning, or rubbing movements. Nocturnal video-EEG monitoring is of foremost importance in order to recognize the different clinical pattern of nocturnal seizures in LGS and r(20) syndrome (69).

### Psychiatric Conditions

The clinical onset of seizures in r(20) consisting of terrific hallucinations and seizures with ictal fears (20, 70, 72) may be confused with different types of visual hallucinations such as the hypnagogic and hypnopompic hallucinations of narcolepsy (84), those of childhood-onset schizophrenia, and others associated with bipolar type-I disorder, major depressive disorder, and other types of psychiatric disorders (85). EEG recordings in the reported psychiatric conditions are normal, and hallucinations are associated with other psychiatric symptoms (e.g., isolation, psychomotor poverty).

Moreover, especially in adolescents, a substance abuse disorder should be considered, and toxicology screen and blood alcohol level should be ordered.

### Autoimmune Diseases

The clinical onset of r(20) syndrome in childhood is often an abrupt constellation of symptoms attributable to brain dysfunction that might recall those of autoimmune encephalopathies. Autoimmune encephalopathies—especially the anti-NMDAR encephalopathy—are characterized at onset by at least four symptoms among: epileptic seizures, movement disorders, psychomotor regression, psychosis, speech dysfunction, memory deficit, sleep disorders, autonomic instability, and decreased consciousness (86). With the exception of movement disorders, autonomic instability, and decreased consciousness, all the other symptoms are found in r(20) syndrome as well. In particular, hyperkinetic seizures are found in both conditions (67, 87). Cognitive impairment and disintegration of language in children are common in both (20, 86). However, the EEG patterns are different in autoimmune encephalopathies (88) and r(20) syndrome, and video EEG monitoring is therefore mandatory for the correct diagnosis.

### Genetic Conditions

Several genetic conditions share neurologic and psychiatric comorbidities together with focal/multifocal epileptic seizures with r(20) syndrome. Among these, patients with 22q13.3 deletion (Phelan-McDermid, OMIM #606232) syndrome present with relatively mild dysmorphic features, ID, psychiatric symptoms, and focal/generalized seizures (89). Although the typical EEG abnormalities seen in patients with Phelan-McDermid syndrome consist of multifocal paroxysmal anomalies that are prevalent over the frontal-central and frontal-temporal regions and are activated during sleep, epilepsy shows a benign course in these patients, unlike r(20) where seizures are usually drug-resistant (89).

Also ring chromosome 14 [r(14)] syndrome is characterized by ID, behavioral changes, and drug-resistant epilepsy with a correlation between age at seizure onset and phenotype severity (55). Status epilepticus is frequent, but seizures usually start in the first months/years of life, unlike in r(20) syndrome. Moreover, individuals with r(14) usually present with a distinctive facial appearance (epicanthic folds, down-slanting palpebral fissures, flat nasal bridge, upturned nares, and large low-set ears) and exhibit ocular manifestations that have never been reported in r(20) syndrome, thus facilitating distinguishing these two conditions (55).

## Future Perspectives

While the phenotype of r(20) syndrome has been extensively delineated and the diagnosis is relatively easy through karyotyping when this condition is suspected, many questions about the mechanism through which ring 20 chromosome causes the typical manifestations remain unanswered.

This depends on the challenge inherent to the mosaic state of r(20) syndrome, which may vary in degree in different tissues, thus limiting the explorative approaches to the most accessible tissues. The survey of genomic results indicates as unlikely the loss of genetic material, as about 50 of the tested affected individuals did not harbor chromosomal deletions (or duplications) detectable by CMA (10, 12, 24, 28, 30, 38, 90, 91).

Whole exome sequencing (WES) has not yet been applied to the study of r(20). It is considered a non-promising approach to yield breakthroughs, though analyzing trios including familial r(20) carriers (mother and offspring) might be a worthwhile exploration. Whole genome sequencing (WGS) may instead be employed to look for complex rearrangements and structural variants overlooked by CMA. This would be consistent with multiple pieces of evidence on altered gene expression upon subtle structural changes affecting the non-coding regulatory genome. Interestingly, subtle structural changes affecting the non-coding regulatory genome may alter the expression of genes.

Alteration of the overall methylation profile has been ruled on a set of 10 patients with r(20) by our data presented herein (Figures 1q,r), but higher resolution of the bead chip and/or customized coverage of the chromosome 20 subtelomeric regions are warranted to confirm these results in the future. Targeted RNA approaches, namely RT-QPCR have been used to assess the expression of genes nearby the fused telomeres in patients with r(14) and r(17) and normal copy number (9, 11) hinting that the clinical phenotype might be ascribed to changes in chromatin architecture. However, whole transcriptome analysis by RNA Sequencing to disclose differentially expressed genes (DEGs) between individuals with r(20) and controls across the entire genome (i.e., long distance effects) has not yet been performed. It is worth noting that transcriptomics approaches are hampered by the availability of the suitable tissue to test. Although several epilepsy-related genes are detectable in blood and previous studies have demonstrated that blood expression analysis is capable of guiding candidate gene identification in neurological disorders (92), gene expression analysis on peripheral blood may lead to inconclusive results and brain-derived tissues may be needed. Based on these considerations, a multi-Omics approach combining genome sequencing and RNA sequencing seems the most-reasonable approach.

Another possible avenue to explore is the employment of iPSCs, in terms of both pathogenesis and therapies. To this regard we may extrapolate to r(20) the result obtained on iPSC generated from fibroblasts of a patient carrying r(17). This *in vitro* model provided enticing insights on the tool of “chromosome therapy” as the unstable behavior of the r(17) during cell division favored emerging aneuploid cells without the ring chromosome and with duplicated wild-type homolog via compensatory UPD (50). The *in vitro* correction of the structurally abnormal chromosome (in the specific case also endowed with a large deletion) raises the challenge of autologous cell mediated therapy for patients (93). However, further basic and translational studies are needed to monitor the potential therapeutic application of these cells (93). For instance, the delivery of patient-specific iPSC-derived neuronal cells to the brain is theoretically feasible, but its efficacy would probably depend on timing (i.e., before or after seizure onset).

So far, the use of iPSCs has been successful in providing an *in vitro* disease model. This is true especially for neurodevelopmental disorders where the neuronal model offers the possibility to explore the pathomechanism of a pathogenic variant in the right context. Unfortunately, this opportunity appears precluded for ring chromosome syndromes, as the RC is lost early after reprogramming and before any iPSC-induced differentiation.

Tackling the r(20) formation and phenotypic consequences remains highly complex, even though iPSC-derived neuronal progenitors maintaining a structurally complete r(20) may provide the system to map its position and folding within the nucleus using multiple methods to decode 3D chromosome architecture (94).

## Conclusions

Ring chromosome 20 syndrome is a rare and likely underdiagnosed condition characterized by a distinctive epileptic phenotype. Difficult-to-treat frontal lobe epilepsy with typical EEG pattern, ID manifesting after seizure onset in an otherwise normally developing child or adolescent with no facial dysmorphisms or birth defects, and behavioral changes are the core clinical manifestations that should lead the neurologist and/or the clinical geneticist to suspect r(20) syndrome (Table 4). We advocate for offering karyotype with 50–100 metaphases count in such cases before requesting other molecular analyses such as CMA and Next Generation Sequencing approaches (i.e., panels, WES or WGS) and whenever these analyses return negative results in patients with overlapping phenotypes of r(20) syndrome (1, 95). Conventional karyotype is a cost-effective and fast test that should not be neglected in the diagnostic approach of patients with these characteristics.

**Table 4:** Practical key points of ring chromosome 20 syndrome.

Core phenotype	• Refractory seizures and frequent non-convulsive status epilepticus (NCSE) are the most common seizure types • Cognitive decline following seizure onset in a previously normally developing child is frequent • Terrific hallucinations are frequent • Growth is usually normal, and dysmorphisms and congenital malformations are uncommon
Inheritance	r(20) occurs sporadically in most patients, but mosaic-to-mosaic transmission has been reported
Diagnosis	Karyotype with high number of metaphase count is the gold standard for diagnosis
Miscellaneous	• Constitutional non-supernumerary r(20) can be mosaic (more frequently) or non-mosaic • In mosaic r(20) the percentage of cells containing the ring chromosome inversely correlates with the age of seizure onset

From a research perspective, new multi-Omics approaches and the use of *in vitro* models and iPSCs may clarify the so far unknown mechanisms through which r(20) causes the clinical manifestations, and ultimately guide the development of new therapies.

## Author Contributions

AP: conceptualized the idea, clinically assessed previously reported patients, reviewed the literature, interpreted the literature review analyses, wrote, assembled, and reviewed the manuscript. IC and MPR: reviewed the literature, performed the literature review analyses, and wrote the manuscript. LC: performed methylation studies. LL: wrote the manuscript. AV and MPC: clinically assessed previously reported patients and wrote the manuscript. All authors critically revised the manuscript for important intellectual content and approved it for submission.

## Conflict of Interest

The authors declare that the research was conducted in the absence of any commercial or financial relationships that could be construed as a potential conflict of interest.
